# A Rare Case of Esophageal Small-Cell Neuroendocrine Carcinoma Presenting With Progressive Dysphagia

**DOI:** 10.7759/cureus.108359

**Published:** 2026-05-06

**Authors:** Ghazal Hindy, Anil Harrison, Sajiv Chandradas

**Affiliations:** 1 Medicine, Midwestern University Arizona College of Osteopathic Medicine, Glendale, USA; 2 Internal Medicine, Midwestern University Arizona College of Osteopathic Medicine, Glendale, USA; 3 Gastroenterology and Hepatology, Scripps Mercy Hospital, San Diego, USA

**Keywords:** dysphagia, endoscopic ultrasound (eus), esophageal malignancy, immunohistochemistry staining, small-cell lung carcinoma, small-cell neuroendocrine tumors

## Abstract

Esophageal neuroendocrine carcinoma (ENEC) is a rare, aggressive cancer that presents with nonspecific symptoms, often mimicking more common esophageal malignancies. We report the case of an 85-year-old man with progressive dysphagia and odynophagia who was found to have a high-grade small-cell neuroendocrine carcinoma (SCNEC) of the mid-esophagus. Diagnosis was confirmed through histopathology and immunohistochemistry (IHC), including synaptophysin, INSM1, and a Ki-67 index near 100%, consistent with a highly proliferative tumor and aggressive biologic behavior. Endoscopic ultrasound (EUS) played a critical role in locoregional staging, demonstrating a T3 lesion without nodal involvement, and providing key information that, together with other clinical factors, informed curative-intent management planning. A multidisciplinary approach enabled timely diagnosis and treatment planning. This case highlights the importance of maintaining a broad differential diagnosis and the value of EUS as an essential tool for accurate staging and management of rare esophageal tumors.

## Introduction

Esophageal neuroendocrine carcinoma (ENEC) is a rare and highly aggressive malignancy with a reported incidence of less than 1% of all esophageal cancers and a tendency for early metastatic spread [1-4]. The World Health Organization (WHO) classifies these tumors as either well-differentiated or poorly differentiated neuroendocrine neoplasms based on morphologic features and proliferative index [1]. They are further categorized into small cell, large cell, or mixed types - the latter containing components of adenocarcinoma or squamous cell carcinoma in addition to neuroendocrine elements and are sometimes referred to as mixed neuroendocrine-non-neuroendocrine neoplasms [1,5].

ENEC is extremely uncommon; one population-based study estimated that small-cell carcinoma accounts for approximately 0.5% to 3.8% of all esophageal cancers [2]. Other population-based analyses similarly report an incidence well below 1% with male predominance and presentation in older adults [3,4]. Its rarity and aggressive nature contribute to challenges in the diagnosis and management and limit the development of prospective randomized trials [3,6]. Clinical presentation often includes dysphagia and weight loss, prompting further diagnostic workup similar to other esophageal malignancies [3,7]. Esophagogastroduodenoscopy (EGD) is typically the initial diagnostic step for the evaluation of suspected esophageal cancer and allows direct visualization and biopsy [7]. However, immunohistochemistry (IHC) is essential to confirm the neuroendocrine subtype and differentiate neuroendocrine carcinoma (NEC) from poorly differentiated squamous cell carcinoma or adenocarcinoma [1,5]. Common neuroendocrine markers include synaptophysin, chromogranin A, INSM1, and CD56, with INSM1 demonstrating high sensitivity for neuroendocrine differentiation [5]. Ki-67 is used to assess tumor proliferation; a Ki-67 index >20% and a mitotic count >20 per mm² are characteristics of poorly differentiated NEC and are associated with aggressive clinical behavior and poor prognosis [1,5].

Due to its rarity, risk factors are not as well characterized as other esophageal malignancies; however, available data suggest significant associations with cigarette smoking and alcohol use, with combined exposure conferring substantially increased risk [8]. In addition, there are no standardized treatment guidelines for ENEC, and management is frequently extrapolated from small-cell lung cancer protocols [6,9]. Management often parallels treatment strategies for small-cell lung cancer, including platinum-based chemotherapy regimens such as cisplatin or carboplatin combined with etoposide [6,9]. Surgery may be considered in select cases with limited-stage disease as part of a multimodal approach [9]. Immunotherapy is an emerging area of interest based on recent retrospective series and extrapolation from small-cell lung cancer literature [10]. Herein, we present a rare case of a localized small- cell NEC in an elderly patient to highlight the diagnostic utility of endoscopic ultrasound (EUS) and the potential for curative-intent management.

## Case presentation

An 85-year-old man presented to the gastroenterology clinic with a two-month history of progressive dysphagia and odynophagia to both solids and liquids. Symptoms began with difficulty swallowing solid foods and later progressed to include liquids. He described a persistent sensation of food sticking in the mid-chest and reported intermittent non-bloody emesis. He denied weight loss, hematemesis, melena, hematochezia, or abdominal pain. His past medical history included gastroesophageal reflux disease (GERD), hypothyroidism, benign prostatic hyperplasia (BPH), and mild normocytic anemia (hemoglobin 12.7 g/dL). He was a former smoker (22-pack-year history, smoking from age 13 to 35 years). Family history was notable for a sister with lung cancer at the age of 85 years, a daughter with breast and renal cancer, and a mother with leukemia at the age of 59 years. Home medications included pantoprazole 40 mg daily, levothyroxine 125 µg daily, and tamsulosin 0.4 mg daily. On examination, the patient appeared well-nourished and in no acute distress. Vital signs were normal. Weight was 200 lbs, height 175 cm, with a body mass index (BMI) of 29.5 kg/m². The remainder of the physical exam was unremarkable.

**Figure 1:**
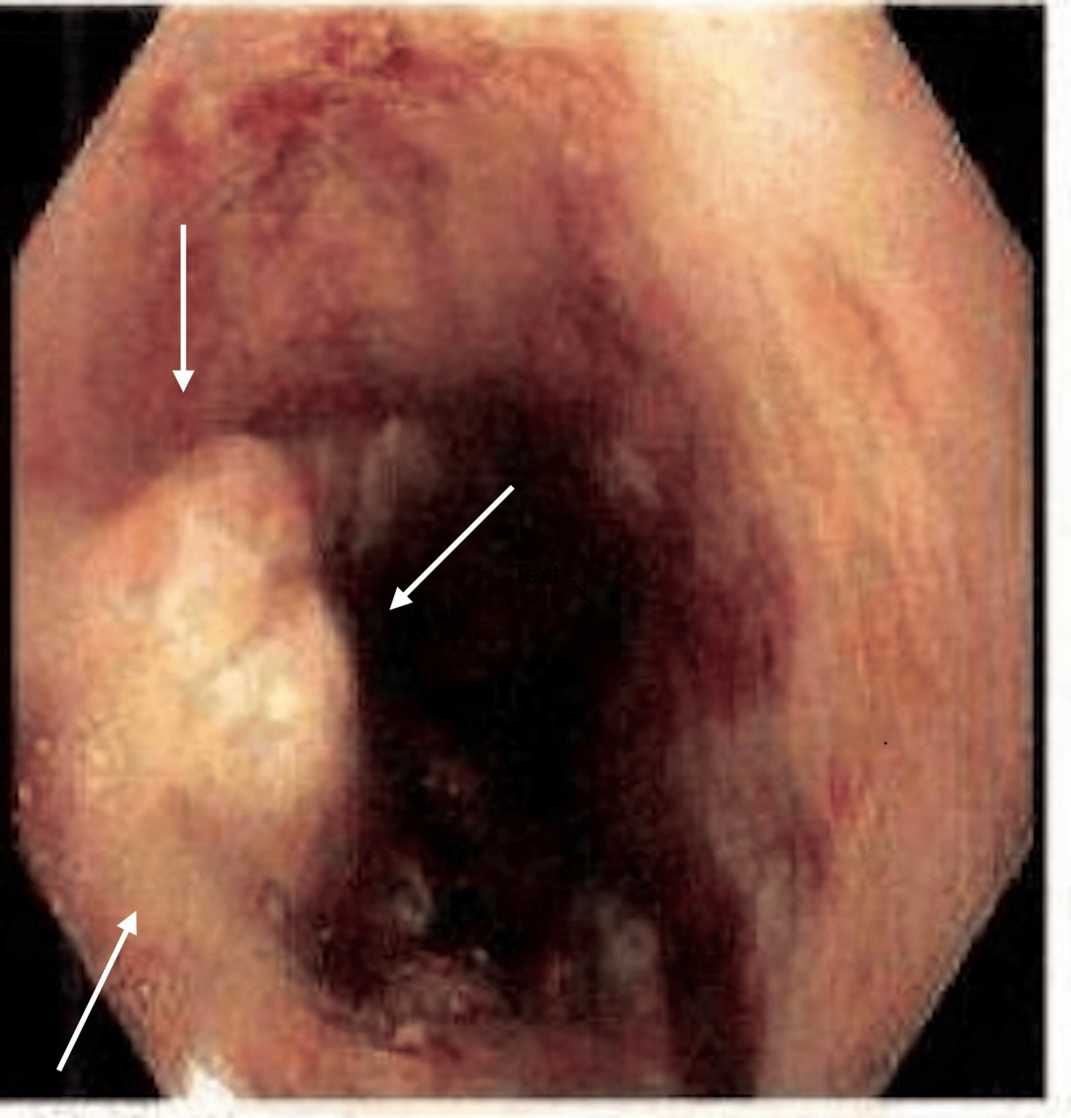
A hemicircumferential ulcerated, fungating, and villous 6 cm mass with stigmata found from 29 cm to 35 cm. The arrows indicate the ulcerated villous mass with stigmata of recent bleeding found in the esophagus.

**Figure 2:**
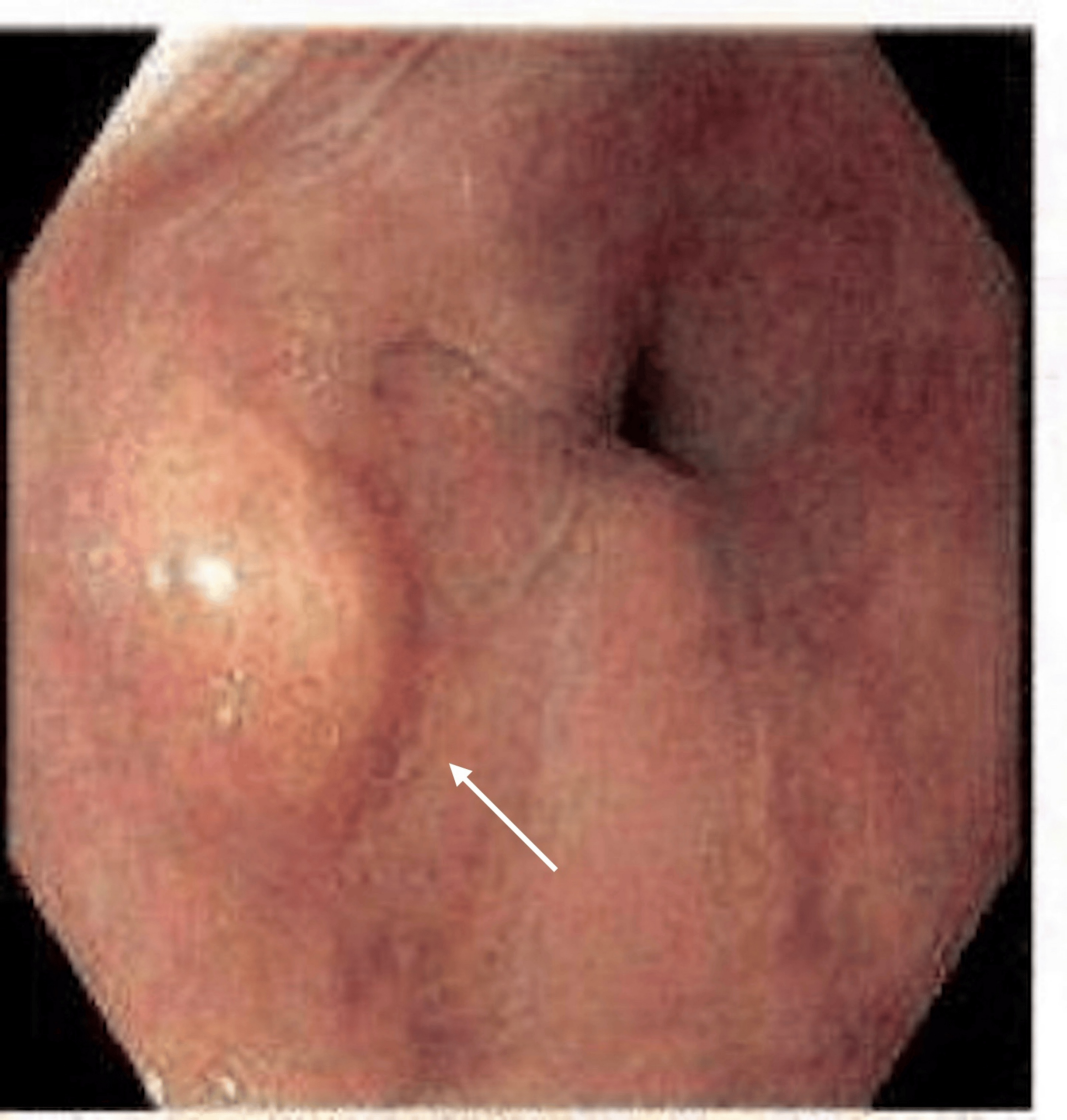
A single localized nodule, sized 8 mm, was observed in the lower esophagus. The arrow indicates a localized nodule in the esophagus.

**Figure 3:**
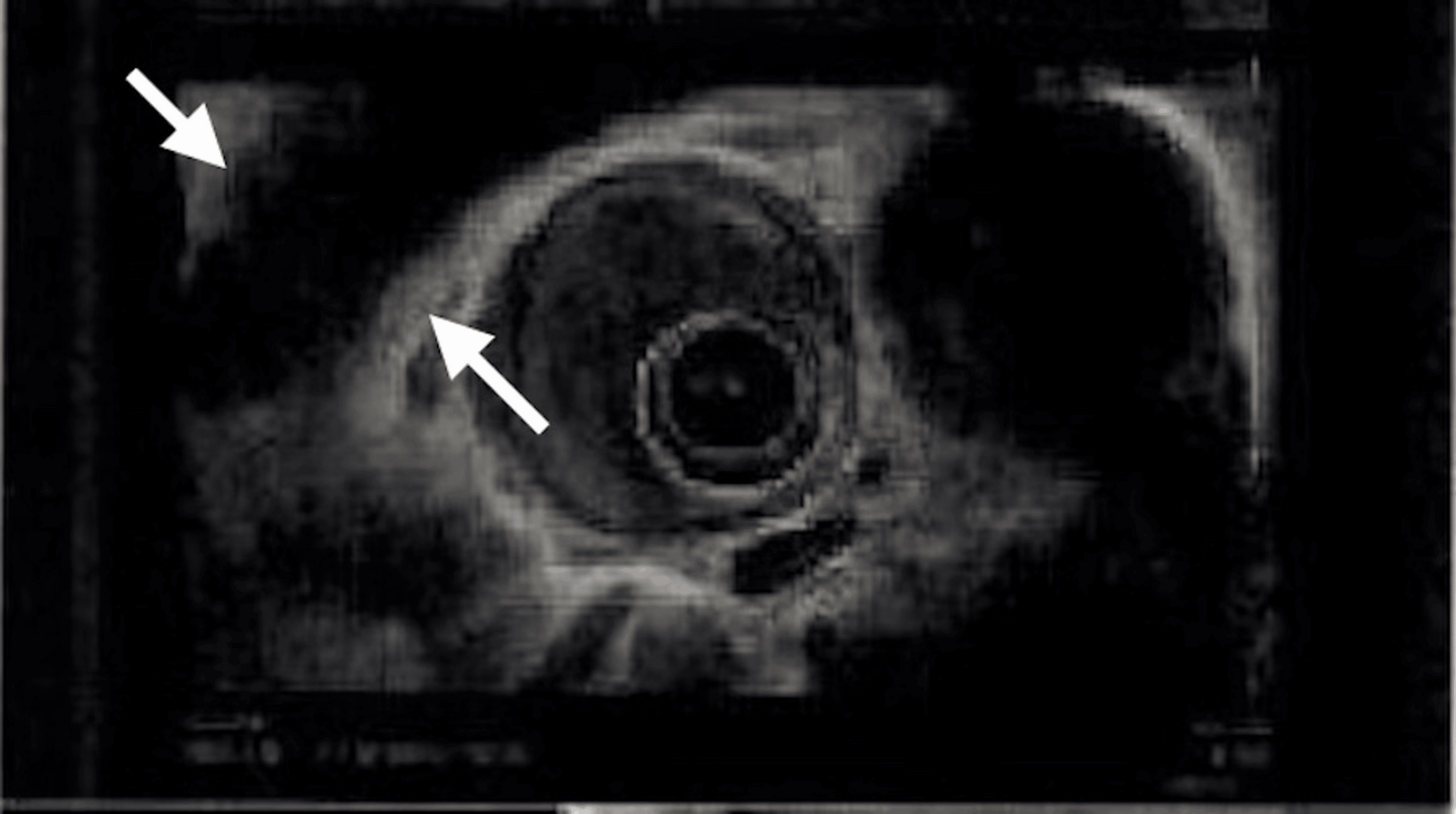
Endosonographic findings confirm a hypoechoic, round, submucosal, and ulcerated T3Nx mass originating in the mucosa and extending to the muscularis propria. The arrows indicate the hypoechoic mass with irregular margins originating in the mucosa and extending into the muscularis propria.

EGD performed five days later revealed an ulcerating, fungating, hemicircumferential mass measuring ~6 cm, located 29-35 cm from the incisors in the mid-esophagus. The lesion appeared malignant with stigmata of recent bleeding and partial luminal obstruction (Figure 1). A second 8 mm nodule was noted at 38 cm, proximal to the gastroesophageal junction at 41 cm (Figure 2). This lesion demonstrated identical histopathologic features to the primary mass and was interpreted as a satellite lesion, consistent with local tumor spread, without impact on overall clinical staging. Biopsies were obtained from both lesions. Histopathology of the esophageal nodule showed a poorly differentiated high-grade NEC, small cell type. Microscopy demonstrated sheets of small hyperchromatic cells with scant cytoplasm and marked nuclear atypia. IHC was positive for pan-cytokeratin (dot-like pattern), INSM1, and synaptophysin, with a Ki-67 proliferation index approaching 100%. S-100 was negative. Biopsies from the mid-esophageal mass showed similar features, confirming the diagnosis. Contrast-enhanced CT of the chest, abdomen, and pelvis showed segmental thickening of the mid-thoracic esophagus without evidence of distant metastases. EUS was crucial for local staging and demonstrated a 40 mm submucosal, ulcerated, hypoechoic mass with irregular margins extending from 32-36 cm, involving approximately three-quarters of the esophageal circumference (Figure 3). No suspicious regional lymphadenopathy was seen, resulting in an initial stage of T3Nx. The Nx reflects the absence of pathological nodal sampling or histologic confirmation of nodal status. 

Final nodal status (N0) was determined based on the absence of suspicious lymph nodes on the CT and EUS findings, thereby allowing definitive pathologic staging according to the AJCC eighth edition criteria. The usage of EUS was instrumental in assessing tumor depth and ruling out locoregional lymph node involvement. A summary of the diagnostic evaluation and staging workup is provided in Table 1.

**Table 1:** Diagnostic evaluation and key findings in small-cell neuroendocrine carcinoma of the esophagus CT: computed tomography

Modality	Findings	Key details
Esophagogastroduodenoscopy (EGD)	Esophageal mass with a distal nodule	A 6 cm fungating, ulcerated mass in the mid-esophagus; additional nodule in the lower third
CT chest/abdomen/pelvis	Esophageal wall thickening	Mid-thoracic circumferential wall thickening
Endoscopic ultrasound (EUS)	Circumferential locally advanced mass	~40 mm hypoechoic submucosal mass involving approximately 75% of the circumference with irregular margins and extension to the muscularis propria
Pathology report of esophageal biopsy taken on EUS	Poorly differentiated high-grade small-cell neuroendocrine carcinoma	Small cell morphology with nuclear molding, frequent mitoses, apoptosis, and crush artifact

Based on clinical, radiographic, endoscopic, and histopathologic findings, the patient was diagnosed with high-grade neuroendocrine (small cell) carcinoma of the thoracic esophagus, staged as T3N0M0 according to the American Joint Committee on Cancer (AJCC) eighth edition staging system. The patient was referred to medical oncology, where treatment options were discussed, including induction chemotherapy with cisplatin and etoposide followed by immunotherapy versus completion of four to six cycles of chemotherapy with consideration of subsequent surgical resection. Given the patient’s limited-stage disease (T3N0M0) and preserved functional status, a curative-intent approach with systemic therapy followed by potential surgical resection was considered appropriate. Despite presenting with progressive dysphagia, the patient did not require immediate local palliative interventions, as he was able to maintain adequate oral intake. Final treatment decisions were to be guided by treatment response and patient preferences, with emphasis on quality-of-life considerations.

## Discussion

Small-cell neuroendocrine carcinoma (SCNEC) of the esophagus is a rare malignancy that histologically resembles small-cell lung cancer and is characterized by small hyperchromatic cells, scant cytoplasm, and high mitotic activity with frequent necrosis and nuclear molding [1,5,6]. The Ki-67 index in this case approached 100%, consistent with a high-grade tumor and reflective of rapid proliferative activity typical of poorly differentiated NEC [1,5].

Prognosis is generally poor, though recent studies suggest outcomes may vary depending on the stage and treatment modality [3]. A 2025 meta-analysis of 24 studies (1,618 patients) reported a median overall survival of 22.5 months, with one-, three-, and five-year survival rates of 76%, 47%, and 34%, respectively [3]. Nodal and distant metastases were independent predictors of poor prognosis, with hazard ratios of 1.44 and 2.68, respectively [3]. Other retrospective series similarly report worse survival in patients presenting with metastatic disease compared with limited-stage tumors [6,9]. Population-based studies demonstrate that a substantial proportion of patients present with stage III or IV disease at diagnosis [2,3].

Presentation often mimics more common esophageal malignancies such as adenocarcinoma and squamous cell carcinoma [3,7]. Symptoms typically include progressive dysphagia and weight loss, with less frequent paraneoplastic syndromes compared with pulmonary small-cell carcinoma [6]. In this case, the patient presented with dysphagia and odynophagia but no systemic symptoms, underscoring the diagnostic challenge associated with this rare entity [3].

Diagnosis relies on tissue biopsy with IHC [1,5]. Positive staining for synaptophysin and INSM1, along with a high Ki-67 index, confirmed the diagnosis. In addition to histopathologic confirmation, EUS is a key modality for staging esophageal malignancies, as it provides high-resolution assessment of tumor invasion depth (T stage) and regional lymph node involvement (N stage), which are critical for prognosis and treatment planning [7]. EUS was valuable for accurate staging in this case, revealing a T3 lesion without nodal involvement. Furthermore, EUS played a pivotal role in precise locoregional staging by accurately assessing tumor depth and evaluating for regional lymph node involvement, which directly influenced clinical decision making and supported a curative-intent approach as EUS is considered the most accurate modality for locoregional staging of esophageal malignancies [7].

Due to its rarity, treatment protocols are extrapolated from small-cell lung cancer [6,9]. Platinum-based chemotherapy (e.g., cisplatin and etoposide) remains first-line therapy [6,9]. Multimodal treatment, including surgery or chemoradiation, may improve outcomes in limited-stage disease [9]. Immunotherapy shows early promise but requires further validation [10]. Published literature describes most patients as presenting with advanced-stage disease, frequently with regional nodal involvement or distant metastases at the time of diagnosis [3,6]. First, despite the tumor’s high proliferative index (Ki-67 approaching 100%), this patient presented with localized disease without nodal or distant metastasis. The absence of nodal involvement in this patient highlights the potential impact of early recognition and comprehensive staging in altering the disease trajectory and enabling consideration of curative-intent therapy [9].

Second, the patient was 85 years old at diagnosis. While ENEC can occur in older adults, aggressive malignancies in this age group often present with advanced disease or are managed palliatively due to comorbidities [3]. In this case, preserved functional status and limited-stage disease allowed consideration of curative intent therapy. This emphasizes that chronological age alone should not preclude aggressive staging and multidisciplinary evaluation when clinically appropriate, particularly in patients with good performance status [9].

Although previous case reports and small series of ENEC, particularly SCNEC, have been described, this case adds to the existing literature by demonstrating that even a poorly differentiated NEC with an extremely high Ki-67 proliferation index can present as localized T3N0M0 disease when identified early through prompt endoscopic evaluation [3]. This report reinforces the importance of maintaining a broad differential diagnosis for dysphagia in elderly patients. It also highlights how early tissue diagnosis, combined with comprehensive EUS staging, may expand the opportunity for potentially curative multimodal therapy in a malignancy traditionally associated with advanced presentation and poor prognosis [3,9]. This case highlights the aggressive nature and diagnostic challenges of primary esophageal SCNEC, emphasizing the importance of early recognition and a comprehensive, multimodal diagnostic approach.

## Conclusions

Esophageal SCNEC is a rare and aggressive malignancy that may present with nonspecific symptoms such as progressive dysphagia. This case demonstrates that even high-grade tumors with a markedly elevated Ki-67 index can present with localized disease when identified early. EUS played a critical role in accurate locoregional staging and guided a curative-intent management strategy. Early endoscopic evaluation, tissue diagnosis, and multidisciplinary planning are important components of care that may help optimize outcomes and could expand opportunities for definitive therapy in selected patients, including elderly individuals with limited-stage disease.
